# G-quadruplexes Mark Sites of Methylation Instability Associated with Ageing and Cancer

**DOI:** 10.3390/genes13091665

**Published:** 2022-09-17

**Authors:** Jonas Rauchhaus, Jenna Robinson, Ludovica Monti, Marco Di Antonio

**Affiliations:** 1 Imperial College London, Chemistry Department, Molecular Science Research Hub, 82 Wood Lane, London W12 0BZ, UK; 2 Imperial College London, Department of Bioengineering, Royal School of Mines, Exhibition Road, London SW7 2AZ, UK; 3 Institute of Chemical Biology, Molecular Science Research Hub, 82 Wood Lane, London W12 0BZ, UK; 4 The Francis Crick Institute, 1 Midland Road, London NW1 1AT, UK

**Keywords:** G-quadruplex, ageing, epigenetics

## Abstract

Regulation of the epigenome is critical for healthy cell function but can become disrupted with age, leading to aberrant epigenetic profiles including altered DNA methylation. Recent studies have indicated that DNA methylation homeostasis can be compromised by the formation of DNA secondary structures known as G-quadruplexes (G4s), which form in guanine-rich regions of the genome. G4s can be recognised and bound by certain methylation-regulating enzymes, and in turn perturb the surrounding methylation architecture. However, the effect G4 formation has on DNA methylation at critical epigenetic sites remains elusive and poorly explored. In this work, we investigate the association between G4 sequences and prominent DNA methylation sites, termed ‘ageing clocks’, that act as *bona fide* dysregulated regions in aged and cancerous cells. Using a combination of *in vitro* (G4-seq) and *in cellulo* (BG4-ChIP) G4 distribution maps, we show that ageing clocks sites are significantly enriched with G4-forming sequences. The observed enrichment also varies across species and cell lines, being least significant in healthy cells and more pronounced in tumorigenic cells. Overall, our results suggest a biological significance of G4s in the realm of DNA methylation, which may be important for further deciphering the driving forces of diseases characterised by epigenetic abnormality, including ageing.

## 1. Introduction

**Figure 1:**
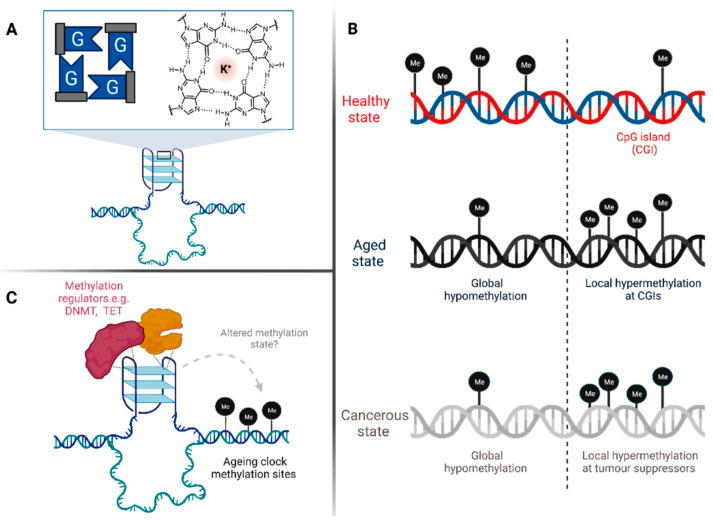
(**A**) Structure of a G4 composed of stacks of guanine quartets. (**B**) Molecular parallels between the methylation changes of aged and cancer cells. (**C**) Model of G4 structures perturbing methylation at ageing clocks sites via interactions with methylation regulators.

How the cell maintains and regulates the expression of its genome has long been a central question of genetic research. However, there is still much to be understood about how the maintenance (or mis-regulation) of the epigenome can influence a cell’s state. Substantial evidence has pointed towards a novel role of DNA secondary structures in epigenetic regulation [1,2,3]. The most well studied of these structures is the G-quadruplex (G4)—a folded state of DNA that arises when guanine(G)-rich sequences assemble into G-quartets—stabilised by Hoogsteen hydrogen bonds and interactions with metal cations such as K^+^ (Figure 1A) [4].

The study of G4s in biology has been substantially accelerated by the development of various methods to map G4s across the genome [5], starting with computational algorithms that use the base composition of a sequence to predict G4-forming propensity [6]. Alternatively, *in vitro* methods, such as G4-seq, identify G4 sequences as genomic regions that result in DNA polymerase stalling under G4-stabilising conditions [7,8]. Finally, to achieve G4 mapping in a cellular context, BG4-ChIP can be employed where a G4-specific antibody (BG4) is used to enrich for G4s formed in chromatin extracted from cells [9,10]. The application of such sequencing methods has revealed associations between G4 formation and various disease states, for example BG4-ChIP data has revealed enhanced G4 enrichment in particular cancer subtypes and cell lines [9,11,12]. Additionally, G4 predictive algorithms have been used to associate G4 sequences with rare, accelerated ageing diseases such as Cockayne’s [13] and Werner’s syndrome [14].

It has been speculated that G4s may contribute to the progression of certain disease states by influencing epigenetic control within cells, which often goes awry in cancerous and aged tissues. Increasing evidence suggests that G4s mediate key DNA–protein interactions, including those of epigenetic modulators such as DNA methyl transferases [1,2,3]. In particular, the interaction of DNA methyltransferase 1 (DNMT1) with G4s has been reported to hinder the normal function of the enzyme, resulting in nearby methylation depletion and potentially altered gene expression [15,16,17]. However, further investigations of G4 formation at genomic sites regulated by DNA methylation are needed to fully disentangle the contribution of G4s to diseases driven by methylation change.

When considering ageing, recent studies have identified key DNA sites—coined *ageing clocks*—for which the methylation state can be used to predict age with impressive accuracy [18,19]. Ageing clocks can be chronological (relating to the actual age of an individual) or biological (indicating a person’s general health and longevity). Interestingly, when the first-ever ageing clock (published by Horvath) was used to calculate the age of 20 cancer cell types, it was found that all cancers displayed accelerated ageing of an average of 36 years compared to healthy cells [19]. Additionally, similar methylation changes occur in both aged cells and cancer cells, including global reductions in DNA methylation, as well as a contrasting increase in methylation at promoters and tumor-suppressor genes (Figure 1B) [20].

Aged and cancer cells thus appear to have multiple molecular parallels, exemplified by similar changes in the methylation landscape, as well as global increases in G4 prevalence particularly in regions associated with abnormal gene expression [9,12,14,21,22]. It is therefore conceivable that enhanced G4 formation within key regulatory sites, such as ageing clocks, may contribute to epigenetic dysregulation in these cells-owing to the disruptive interactions of G4s with methylation regulators such as DNMT1 (Figure 1C) [14]. This hypothesis is further supported by the reported enrichment of putative G4 sequences around the Horvath ageing clock [23], again indicating a potential role of G4s in age-related epigenetic instability.

However, studies investigating the correlation between G4 formation at ageing clock sites and ageing or cancer development are extremely limited. For example, it is yet to be examined if the enrichment of G4 sequences around the Horvath ageing clock is also observed with other ageing clock coordinates more recently developed in humans and other species. Moreover, previous work has relied on computational approaches to predict the G4-forming ability of a sequence [23], which does not consider the chromatin context of DNA that influences both DNA methylation and the ability of a sequence to actually fold into a G4 structure within the nucleus. This means that *in silico* predictions of G4 formation cannot be used to confidently evaluate cellular G4 formation, or to investigate how G4 prevalence varies for different cell types.

In this study, we aim to expand the current understanding of how G4s are connected to methylation regulation by investigating the colocalization of G4s with various biological and chronological ageing clocks in humans and mice. Using G4-seq data, we first show that G4s are substantially more enriched at the ageing clocks of humans compared to mice, which is coupled with an enhanced colocalization of G4s with methylation regulators such as DNMT and TET enzymes. Furthermore, we used BG4-ChIP data to assess how such enrichment of G4s at ageing clocks varies between cell lines with various levels of genomic instability. Specifically, we consider oncogenic cell lines to be a suitable model for investigating epigenetic dysfunction, owing to the analogous methylation changes of cancer and aged cells. Our analysis shows that G4 enrichment becomes significantly more pronounced at ageing clocks when moving from healthy to tumorigenic cells. These findings suggest that G4s mark sites where epigenetic changes commonly accumulate with age and may thus have a determining role on epigenetic stability within cells. Further investigation of G4 formation around ageing clocks may therefore be important to better understand, and ultimately reverse, the epigenetic drift that drives many age-related conditions.

## 2. Materials and Methods

All analyses were conducted in R version 4.0.3.

All genomic coordinates were converted to the hg19 (human) or mm10 (mouse) genome build before analysis using the *liftOver* function of the *rtracklayer* package.

### 2.1. Enrichment and Statistical Tests

#### 2.1.1. Window Size Analysis

**Figure 2:**
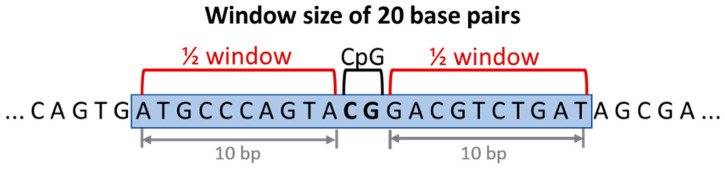
Creation of windows around CpG coordinates.

To assess how G4 enrichment around ageing clocks is affected by distance, enrichment was calculated within various window sizes surrounding each ageing clock CpG coordinate. The start and end coordinates of each CpG site was extended by half of the given window size (Figure 2), for a total of 10–1,000,000 bp.

#### 2.1.2. G4 Enrichment Relative to Random

To evaluate the degree to which G4s colocalise with ageing clock sites, G4 coordinates were intersected with ageing clock coordinates (extended by a given window size) using the *intersect* function of the GenomicRanges package in *R*. The G4-file was then randomized 30-times using the *randomizeRegions* function of the *regionR* package, to create 30 files with the same number of features, but randomly shuffled coordinates. The shuffled G4 files were intersected with ageing clock coordinates. Fold-enrichment of G4s at ageing clocks (F) was calculated as:

$$F=\frac{c}{1+t}$$

where *c* is the number of base pairs that overlap between the G4 and ageing clock files and *t* is the average number of overlaps between the shuffled G4 and ageing clock files. A constant of 1 was introduced to the denominator to account for cases where the average number of random overlaps was <1.

To statistically test the results of the enrichment, an upper, one-tailed Z-test was performed using the *pnorm* command from the R stats package. The number of overlaps from the shuffled-file intersections were assumed to be normally distributed and thus acted as the expected population distribution under the null hypothesis. This test yielded a *p*-value where an enrichment was said to be statistically significant if *p* < 0.01.

#### 2.1.3. G4 Enrichment Relative to Global CpG Enrichment

The second described enrichment test considers the enrichment of G4s around ageing clock CpGs relative to the enrichment of G4s around all CpGs genome-wide:

1. All instances of the CG motif were extracted from the reference genomes using the function matchPattern(‘CG’, chromosome) from the Biostrings package;
2. The coordinates of CpGs that overlap with G4s were found using *intersect* from the GenomicRanges package;
3. The fraction (f) of CpGs that intersect with G4s was found by applying *subsetByOverlaps* to the intersected file;
4. The expected number (E[n]) of ageing clock CpGs intersecting with G4s was found by multiplying f by the total number of ageing clock CpGs in the genome;
5. The actual number (n) of G4s intersecting with ageing clock CpGs was calculated as before using *intersect* and *subsetByOverlap* applied to the respective ageing clock files;
6. The fold-enrichment of G4s at ageing clock CpGs relative to the expected from all CpGs was then found as n/E[n].

A hypergeometric test conducted using the *phyper* function was used to test for the statistical significance of this enrichment, where an enrichment was said to be statistically significant if *p* < 0.01.

#### 2.1.4. G4 Enrichment at Protein-Binding Sites

G4 enrichment at the binding sites of methylation-regulating proteins DNMT and TET were found using an analogous method as detailed in the ‘relative to random’ enrichment test, where ChIP-seq peaks of proteins were used in place of extended CpG coordinates.

## 3. Results

### 3.1. G4 Sequences Co-Localise with Chronological and Biological Ageing Clocks in Humans

To begin investigating G4 prevalence around ageing clocks, we utilised previously published G4-seq data, which define putative G4 coordinates across the human genome leveraging *in vitro* polymerase-stalling experiments [7,8]. We first considered the extent to which G4 sequences overlap with two human ageing clocks: (i) the Horvath clock that predicts chronological age [19] and (ii) the Levine clock that predicts general biological function and longevity [24]. Both clocks define CpG (cytosine–phosphate–guanine) sites that change in methylation state significantly with age, as determined by a multi-tissue analysis. To identify overlaps between the ageing clocks and G4s, the individual CpG coordinates from the clocks were extended by various window sizes (10–1,000,000 bp) and then intersected with the coordinates of the G4-seq dataset. To test for statistical significance, the ageing clock coordinates were also intersected with a “shuffled” G4-seq dataset where G4 coordinates were randomly shuffled throughout the genome, allowing us to compare the number of genuine G4 overlaps against the number of expected random overlaps.

**Figure 3:**
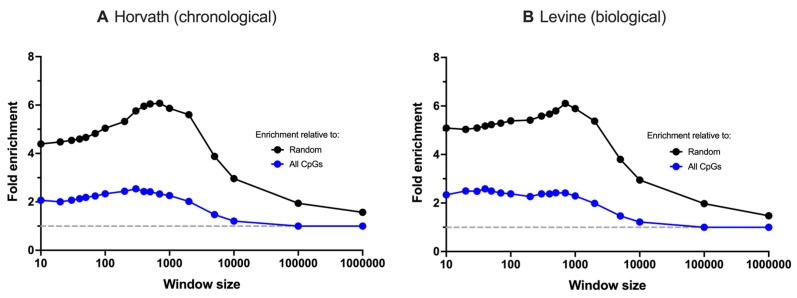
Enrichment of human G4 sequences at (**A**) Horvath chronological ageing clock and (**B**) Levine biological ageing clock. Curves are for enrichment relative to randomly shuffled G4 coordinates (black) or relative to enrichment of G4s at CpGs globally (blue). Dotted grey line at fold-enrichment = 1 for reference.

From our analysis, we found that G4 sequences were significantly more likely to intersect with regions around ageing clock CpGs compared to randomly shuffled coordinates, with an enrichment of up to six-fold (Figure 3, Supplementary Figure S1). The noted enrichment could be seen for a window size of just 10 bp around a CpG site (~four-fold enrichment over random), with increasing enrichment up to a 1000 bp window (~six-fold enrichment, *p <* 10^−200^). This demonstrates that whilst G4s colocalise with ageing clocks proximally, G4 enrichment is highest at larger distances of around 1000 bp. It has been previously proposed that G4s may act as protein binding hubs, restraining methylation-regulating proteins away from local CpG sites [14]. It is thus plausible that G4s may have more pronounced effects on methylation regulation when located somewhat distally from key CpG sites where they can sequester regulatory proteins out of reach of the dysregulated CpG.

We additionally considered if G4 enrichment varied between the chronological and biological ageing clocks. We found that both the Horvath and Levine clock have comparable levels of G4 enrichment, with both showing maximum enrichment at around 1000 bp. Interestingly, previous analysis revealed that the two ageing clocks display limited overlap, with just 41 of the 513 CpGs that make up the Levine clock (~8%) being present in the Horvath clock [21]. The shared G4 enrichment at both clocks may thus indicate a general selection and role of G4s around DNA regions that are epigenetically dysregulated with ageing and declining health.

We noted that a potential explanation for the high G4 enrichment at ageing clocks is the non-random distribution of CpG sites across the genome. As CpGs are often found clustered together into CpG islands (CGIs), it is expected that many CpGs exist in regions that are particularly high in guanine content and thus more likely to form G4s. Additionally, CpGs are enriched at promoter regions, which are one of the most enriched *loci* for G4 formation [8]. To account for this, we extended our analysis by considering the enrichment of G4s around ageing clock CpGs, relative to the natural enrichment of G4s around CpG regions globally (Figure 3, Supplementary Figure S1). Whilst including this control reduced the absolute fold-enrichment at ageing clocks compared to random, the prevalence of G4s at age-related regions was still significantly higher than expected from chance (up to 2.5-fold enrichment, *p <* 10^−30^). These results demonstrate that G4s, whilst naturally enriched around CpGs globally, are particularly prevalent around ageing clock CpGs, thus supporting a model where G4s may play an active role in regulating age-related DNA-methylation changes.

In addition to the general enrichment of G4s around all ageing clock CpG coordinates, we also considered whether G4s are specifically associated with regions that increase (hyper-) or decrease (hypo-) in methylation with age (Supplementary Figure S2). In the Horvath chronological clock, there was a marginally greater association of G4s at CpGs that decreased in methylation, whereas analysis of the Levine biological clock revealed the opposite trend. However, there was not a significant difference in G4 prevalence at either hyper- or hypo-methylated regions. This result contrasts with previous work suggesting that G4 formation is globally accompanied by local hypomethylation [15] and suggests that the presence of G4s at ageing clocks may have more complex effects on the methylation state, creating areas of general epigenetic instability rather than particularly increased or decreased methylation (Table 1).

### 3.2. Associations between G4s, Ageing Clocks, and Methylation Regulators Are Specific to Humans

**Figure 4:**
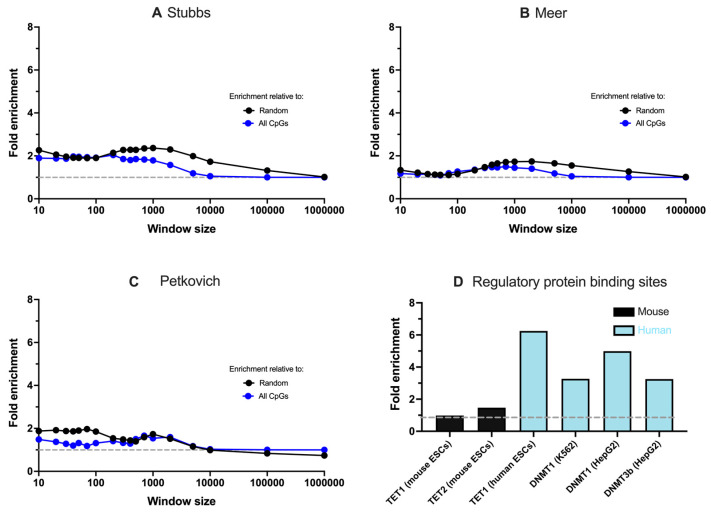
Enrichment of murine G4 sequences at chronological ageing clocks developed by (**A**) Stubbs (**B**) Meer, and (**C**) Petkovich. (**D**) Enrichment of G4-sequences at various TET and DNMT protein binding sites in humans and mice.

To gain further insight into the potential role of G4s in age-related DNA methylation regulation, we considered if correlations between G4 formation and ageing clock coordinates could be observed in other mammalian species, namely mice. The same analysis of enrichment was thus conducted using murine G4-seq data [8] and coordinates from three murine chronological ageing clocks published by Stubbs [22], Petkovich [24], and Meer *et al.* [23]. The G4 enrichment at murine ageing clocks was found to only be statistically significant for a portion of window sizes and this enrichment was notably lower than that seen in humans, with absolute enrichment relative to random being ~2.5-fold compared to the 6-fold enrichment found in humans (Figure 4, Supplementary Figure S3). Similarly, the enrichment relative to global CpG enrichment was generally lower than that seen in humans (1–2-fold; Figure 4, Supplementary Figure S3). A reduced enrichment at murine ageing clocks was found even when a fraction of murine G4 sequences were randomly sampled to obtain comparable numbers to the human G4-seq dataset (Supplementary Table S1).

It is plausible that the reduced enrichment of G4s around the ageing clocks of mice is a consequence of the varied prevalence of G4s at regulatory regions within different organisms. Previous analysis of G4-seq data across multiple species revealed that humans have the highest enrichment of G4s at regulatory sites such as promoters, transcription start sites, and 5′ untranslated regions, which is indicative of a more pronounced regulatory role of G4s in humans [8]. To further test this hypothesis, we used available ChIP-Seq datasets to evaluate the enrichment of G4 sequences amongst binding sites of various proteins that regulate methylation in both humans and mice. This analysis included variants of DNMT enzymes that catalyse the addition of methyl marks to DNA [28]. In addition to this, we analysed the binding sites of TET proteins responsible for the hydroxymethylation of methyl-cytosine bases, which represents one mechanism by which methyl marks are removed from DNA [25,26,27].

From our enrichment analysis, we found that G4 sequences are more frequently associated with the binding sites of methylation-editing proteins in humans than in mice. For instance, G4s were not enriched within the binding sites of TET1 in mice (fold enrichment = 0.98) and were marginally enriched within mouse TET2 protein binding sites (fold enrichment = 1.47) (Figure 4D). Conversely, G4s were significantly enriched, more than six-fold, within the binding sites of TET1 in humans. Furthermore, G4 sequences were significantly enriched (upwards of three-fold) within all the tested human DNMT binding sites, including DNMT1 and DNTMT3b. It is possible that enhanced enrichment of G4s at human protein binding sites may perturb the action of key methylation regulators, which would in turn explain why G4s are more likely to colocalise with epigenetically dysregulated sites in humans than in mice. 

### 3.3. G4 Enrichment at Ageing Clocks Is Greatest in Immortalised and Tumorigenic Cells

A key point of interest of the observed G4 enrichment around ageing clocks, is the connection of age-related dysregulation with disease states such as cancer. As cancer cells exhibit increased epigenetic ageing (according to their DNA methylation profile) [19], they may act as a suitable model to investigate whether G4 prevalence at ageing clocks changes as cells develop epigenetic dysfunctions. We thus considered G4 formation within individual cell lines that display varying degrees of cancer characteristics. Specifically, we analysed BG4-ChIP data, obtained using the G4-specific antibody BG4, which is used to map G4s that are found in chromatin extracted from cells. Our analysis included published BG4-ChIP data on three cells lines: 1. NHEK—normal human epidermal keratinocytes [9]; 2. HaCaT—spontaneously immortalised human skin cells, which have cancer characteristics in terms of proliferation, but are non-tumorigenic [9]; and 3. K562—patient-derived and tumorigenic leukaemia cells [15].

**Figure 5:**
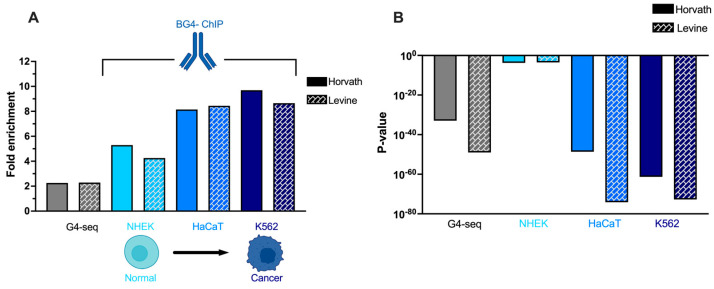
**(A**) Enrichment (relative to global CpG enrichment) of human G4s within 1000 bp of Horvath and Levine ageing clocks sites including enrichment from G4-seq data, as well as cell-based BG4-ChIP experiments in NHEK, HaCaT, and K562 cells. (**B**) Statistical significance of G4 enrichment within 1000 bp of Horvath and Levine ageing clocks found with G4-seq data and in NHEK, HaCaT, and K562 cells.

By applying the same G4 enrichment analysis used for the G4-seq dataset, we found that the enrichment of G4s around ageing clocks, relative to randomly shuffled coordinates, ranged from 35 to 75-fold across the three cells lines, with the highest enrichment being in the cancer cell line K562 (Supplementary Figures S4 and S5). The evaluation of G4 enrichment around ageing clock CpGs, relative to global CpG enrichment, revealed that the enrichment was again markedly higher than that calculated using G4-seq data (Figure 5A). This suggests that, of all the putative G4 sequences identified by G4-seq, those sequences near ageing clocks are particularly likely to form in cells, further indicating a cellular function of G4s at these regions. Furthermore, the elevated enrichment is potentially due to G4s forming predominantly within open chromatin regions, which also tend to colocalise with regulatory sites such as ageing clocks [9].

Considering the differences between the evaluated cell lines, we found a substantial increase in G4 enrichment when moving from the normal NHEK cells to the cancer-like HaCaT cells and tumorigenic K562 cells. Notably, whilst NHEK cells displayed an enrichment of G4s around ageing clocks, this result should be viewed with caution as statistical analysis yielded a significantly higher *p*-value than that obtained in other cell lines (Figure 5B, Supplementary Figures S4 and S5). In fact, the NHEK cells were the only human cell line that did not reach a significance level of *p* *<* 0.01 for all window sizes. This may be due to the smaller number of G4 peaks obtained in the NHEK line (ca. 1000 peaks in NHEK vs. 10,000 in HaCaT cells) reducing statistical power. Nevertheless, the reduced statistical significance means that the enrichment of G4s around ageing clocks within normal cells cannot be considered as statistically or potentially biologically significant.

Conversely, both the immortalised (HaCaT) and tumorigenic (K562) cell lines showed statistically significant G4 enrichment at all window sizes, according to both enrichment tests and with both ageing clocks. These results demonstrate that G4 prevalence around ageing clock CpGs can vary in a manner that is cell-type specific, being most enriched in cancer-like cells that also display elevated ageing in terms of methylation state. Together, these findings demonstrate that G4 formation at ageing clocks increases with epigenetically aged phenotypes, which may indicate a role of such DNA secondary structures in driving the epigenetic dysfunction that characterises the aged genome.

## 4. Discussion

In this study, we conducted a bioinformatic analysis to investigate the association between G4-forming sequences and genomic ageing clock sites where epigenetic drift associated with ageing and tumorigenesis occurs. We observed a ~six-fold enrichment of human G4 structures amongst both biological and chronological ageing clock coordinates, using an experimentally validated G4-map (G4-Seq). Notably, such enrichment was significantly increased when the same analysis was performed using ChIP-Seq data (BG4-ChIP), which provides a distribution of G4 structures that are formed in a chromatin context. Our analysis also revealed an increased enrichment of G4s at ageing clocks sites when analyzing BG4-ChIP data derived from cancer cell lines, suggesting a link between tumorigenesis and G4 formation at these key regulatory sites.

Importantly, we found that the enrichment of G4s around human ageing clocks is higher than that reported at other key regulatory regions of the human genome. For example, using the same approach, a previous study reported a four-fold enrichment of G4s within 1000 bp of transcription start sites (TSS) [8], which reinforces the hypothesis that G4s are important regulatory elements. Furthermore, significant evidence suggests that the high prevalence of G4s around TSS has measurable biological effects, including elevated gene transcription [3]. Given that our analysis revealed a six-fold enrichment of G4s within 1000 bp of ageing clock sites, it is plausible that G4 enrichment at these key methylation sites may have analogous biological effects, which are yet to be fully elucidated.

It is possible that G4s may influence methylation regulation at ageing clocks through their interactions with epigenetic modifiers, such as TETs and DNMTs, which we found were significantly associated with G4 regions in human cells. The elevated enrichment of G4s amongst binding sites of methylation-regulators in humans suggests that G4 formation may have more relevance for epigenetic regulation in humans compared to other species, which is worth consideration given the frequent use of mouse models to study age-related diseases in humans [29]. Additionally, the comparable enrichment of G4s amongst sites bound by enzymes that both add (DNMTs) and remove (TETs) methylation marks supports a model where G4 formation may generally perturb DNA-methylation, without necessarily promoting either hyper- or hypomethylation.

Altogether, our data reinforce the concept that G4 structures may act as epigenetic regulatory elements and suggests that their formation is associated with the epigenetic drift that contributes to ageing and tumor formation.

## Figures and Tables

**Table 1:** Details of all datasets used in this study.

Data Description	Data Location	Reference
Horvath ageing clock	Publication suppl. data	Horvath (2013) [19]
Levine ageing clock	Publication suppl. Data	Levine et al. (2018) [24]
Stubbs ageing clock	GEO: GSE93957/publication suppl. Data	Stubbs et al. (2017) [25]
Meer ageing clock	GEO: GSE121141/publication suppl. Data)	Meer et al. (2018) [26]
Petkovich ageing clock	GEO: GSE80672/publication suppl. Data)	Petkovich et al. (2017) [27]
Human G4-seq (K^+^)	GEO: GSM3003539	Marsico et al. (2019) [8]
Mice G4-seq (K^+^)	GEO: GSM3003547	Marsico et al. (2019) [8]
TET1 (mouse ESCs) ChIP-Seq	GEO: GSE24841	Williams et al. (2011) [25]
TET2 (mouse ESCs) ChIP-seq	GEO: GSE41720	Chen et al. (2013) [26]
TET1 (human ESCs) ChIP-seq	GEO: GSE99346	Verma et al. (2018) [27]
DNMT1 (K562) ChIP-seq	GEO: GSE92213	ENCODE [28]
DNMT1 (HepG2) ChIP-seq	GEO: GSE170872	ENCODE [28]
DNMT3b (HepG2) ChIP-seq	GEO: GSE95953	ENCODE [28]
NHEK BG4-ChIP	GEO: GSE76688	Hänsel-Hertsch et al. (2016) [9]
HaCaT BG4-ChIP	GEO: GSE76688	Hänsel-Hertsch et al. (2016) [9]
K562 BG4-ChIP	GEO: GSE107690	Mao et al. (2018) [15]

## Data Availability

Code used for analyses reported in this paper can be found at the following github page: https://github.com/ImperialCollegeLondon/G4s_ageing_clocks.

## Supplementary Materials

The following supporting information can be downloaded at: https://www.mdpi.com/article/10.3390/genes13091665/s1, Figure S1: *p*-values for enrichment tests of G4s at Horvath and Levine human ageing clocks; Figure S2: Enrichment of G4s at Horvath and Levine ageing clock CpGs that become hyper- or hypo-methylated with age; Figure S3: *p*-values for enrichment tests of G4s at Stubbs, Meer and Petkovich ageing clocks; Figure S4: Fold enrichment and statistical significance of G4s around Horvath ageing clock CpGs in NHEK, HaCaT, and K562 cells; Figure S5: Fold enrichment and statistical significance of G4s around Levine ageing clock CpGs in NHEK, HaCaT, and K562 cells. Table S1: Comparison of G4 enrichment at murine ageing clocks with and without sampling.

## References

[1] Mukherjee A.K., Sharma S., Chowdhury S. (2019). Non-duplex G-Quadruplex Structures Emerge as Mediators of Epigenetic Modifications. Trends Genet..

[2] Varizhuk A., Isaakova E., Pozmogova G. (2019). DNA G-Quadruplexes (G4s) Modulate Epigenetic (Re)Programming and Chromatin Remodeling. BioEssays.

[3] Robinson J., Raguseo F., Nuccio S.P., Liano D., Di Antonio M. (2021). DNA G-quadruplex structures: More than simple roadblocks to transcription?. Nucleic Acids Res..

[4] Spiegel J., Adhikari S., Balasubramanian S. (2020). The Structure and Function of DNA G-Quadruplexes. Trends Chem..

[5] Raguseo F., Chowdhury S., Minard A., Di Antonio M. (2020). Chemical-biology approaches to probe DNA and RNA G-quadruplex structures in the genome. Chem. Commun..

[6] Puig Lombardi E., Londoño-Vallejo A. (2020). A guide to computational methods for G-quadruplex prediction. Nucleic Acids Res..

[7] Chambers V.S., Marsico G., Boutell J.M., Di Antonio M., Smith G.P., Balasubramanian S. (2015). High-throughput sequencing of DNA G-quadruplex structures in the human genome. Nat. Biotechnol..

[8] Marsico G., Chambers V.S., Sahakyan A.B., McCauley P., Boutell J.M., Di Antonio M., Balasubramanian S. (2019). Whole genome experimental maps of DNA G-quadruplexes in multiple species. Nucleic Acids Res..

[9] Haensel-Hertsch R., Beraldi D., Lensing S.V., Marsico G., Zyner K., Parry A., Di Antonio M., Pike J., Kimura H., Narita M. (2016). G-quadruplex structures mark human regulatory chromatin. Nat. Genet..

[10] Haensel-Hertsch R., Spiegel J., Marsico G., Tannahill D., Balasubramanian S. (2018). Genome-wide mapping of endogenous G-quadruplex DNA structures by chromatin immunoprecipitation and high-throughput sequencing. Nat. Protoc..

[11] Kosiol N., Juranek S., Brossart P., Heine A., Paeschke K. (2021). G-quadruplexes: A promising target for cancer therapy. Mol. Cancer.

[12] Liano D., Chowdhury S., Di Antonio M. (2021). Cockayne Syndrome B Protein Selectively Resolves and Interact with Intermolecular DNA G-Quadruplex Structures. J. Am. Chem. Soc..

[13] Johnson J.E., Cao K., Ryvkin P., Wang L.-S., Johnson F.B. (2010). Altered gene expression in the Werner and Bloom syndromes is associated with sequences having G-quadruplex forming potential. Nucleic Acids Res..

[14] Mao S.-Q., Ghanbarian A.T., Spiegel J., Cuesta S.M., Beraldi D., Di Antonio M., Marsico G., Haensel-Hertsch R., Tannahill D., Balasubramanian S. (2018). DNA G-quadruplex structures mold the DNA methylome. Nat. Struct. Mol. Biol..

[15] Halder R., Halder K., Sharma P., Garg G., Sengupta S., Chowdhury S. (2010). Guanine quadruplex DNA structure restricts methylation of CpG dinucleotides genome-wide. Mol. BioSyst..

[16] Cree S.L., Fredericks R., Miller A., Pearce F.G., Filichev V., Fee C., Kennedy M.A. (2016). DNA G-quadruplexes show strong interaction with DNA methyltransferases in vitro. FEBS Lett..

[17] Bell C.G., Lowe R., Adams P.D., Baccarelli A.A., Beck S., Bell J.T., Christensen B.C., Gladyshev V.N., Heijmans B.T., Horvath S. (2019). DNA methylation aging clocks: Challenges and recommendations. Genome Biol..

[18] Horvath S. (2013). DNA methylation age of human tissues and cell types. Genome Biol..

[19] Johnson A.A., Akman K., Calimport S.R.G., Wuttke D., Stolzing A., de Magalhães J.P. (2012). The Role of DNA Methylation in Aging, Rejuvenation, and Age-Related Disease. Rejuv. Res..

[20] Malousi A., Andreou A.-Z., Georgiou E., Tzimagiorgis G., Kovatsi L., Kouidou S. (2018). Age-dependent methylation in epigenetic clock CpGs is associated with G-quadruplex, co-transcriptionally formed RNA structures and tentative splice sites. Epigenetics.

[21] Levine M.E., Lu A.T., Quach A., Chen B.H., Assimes T.L., Bandinelli S., Hou L., Baccarelli A.A., Stewart J.D., Li Y. (2018). An epigenetic biomarker of aging for lifespan and healthspan. Aging.

[22] Stubbs T.M., Bonder M.J., Stark A.K., Krueger F., von Meyenn F., Stegle O., Reik W., Bolland D., Butcher G., Chandra T. (2017). Multi-Tissue DNA Methylation Age Predictor in Mouse. Genome Biol..

[23] Meer M.V., Podolskiy D.I., Tyshkovskiy A., Gladyshev V.N. (2018). A Whole Lifespan Mouse Multi-Tissue DNA Methylation Clock. eLife.

[24] Petkovich D.A., Podolskiy D.I., Lobanov A.V., Lee S.-G., Miller R.A., Gladyshev V.N. (2017). Using DNA Methylation Profiling to Evaluate Biological Age and Longevity Interventions. Cell Metab..

[25] Williams K., Christensen J., Pedersen M.T., Johansen J.V., Cloos P.A.C., Rappsilber J., Helin K. (2011). TET1 and Hydroxymethylcytosine in Transcription and DNA Methylation Fidelity. Nature.

[26] Chen Q., Chen Y., Bian C., Fujiki R., Yu X. (2013). TET2 promotes histone O-GlcNAcylation during gene transcription. Nature.

[27] Verma N., Pan H., Doré L.C., Shukla A., Li Q.V., Pelham-Webb B., Teijeiro V., González F., Krivtsov A., Chang C.-J. (2017). TET proteins safeguard bivalent promoters from de novo methylation in human embryonic stem cells. Nat. Genet..

[28] Dunham I., Kundaje A., Aldred S.F., Collins P.J., Davis C.A., Doyle F., Epstein C.B., Frietze S., Harrow J., Kaul R. (2012). An Integrated Encyclopedia of DNA Elements in the Human Genome. Nature.

[29] Mitchell S.J., Scheibye-Knudsen M., Longo D.L., de Cabo R. (2015). Animal Models of Aging Research: Implications for Human Aging and Age-Related Diseases. Annu. Rev. Anim. Biosci..

