# Supplementary material for: G-quadruplexes Mark Sites of Methylation Instability Associated with Ageing and Cancer
Source: Genes (Basel). 2022 Sep 17;13(9):1665. doi: 10.3390/genes13091665 (PMC9498706; doi:10.3390/genes13091665)
Supplement: Supplementary file 1 [file genes-13-01665-s001.zip › genes-1848229-supplementary.pdf]

G-quadruplexes mark sites of methylation instability associated with ageing and cancer

Jonas Rauchhaus [1,2†], Jenna Robinson[1,3,4†], Ludovica Monti[1,4] and Marco Di Antonio.[1,3,4*]

[1] Imperial College London, Chemistry Department, Molecular Science Research Hub, 82 Wood Lane, W12 0BZ, London, UK.
[2] Imperial College London, Department of Bioengineering, Royal School of Mines, Exhibition Road, SW7 2AZ, London, UK.
[3] Institute of Chemical Biology, Molecular Science Research Hub, 82 Wood Lane, W12 0BZ, London, UK.
[4] The Francis Crick Institute, 1 Midland Road, NW1 1AT, London, UK.
* Correspondence should be addressed to Marco Di Antonio: m.di-antonio@imperial.ac.uk

† Authors contributed equally to the work.

Supplementary Materials

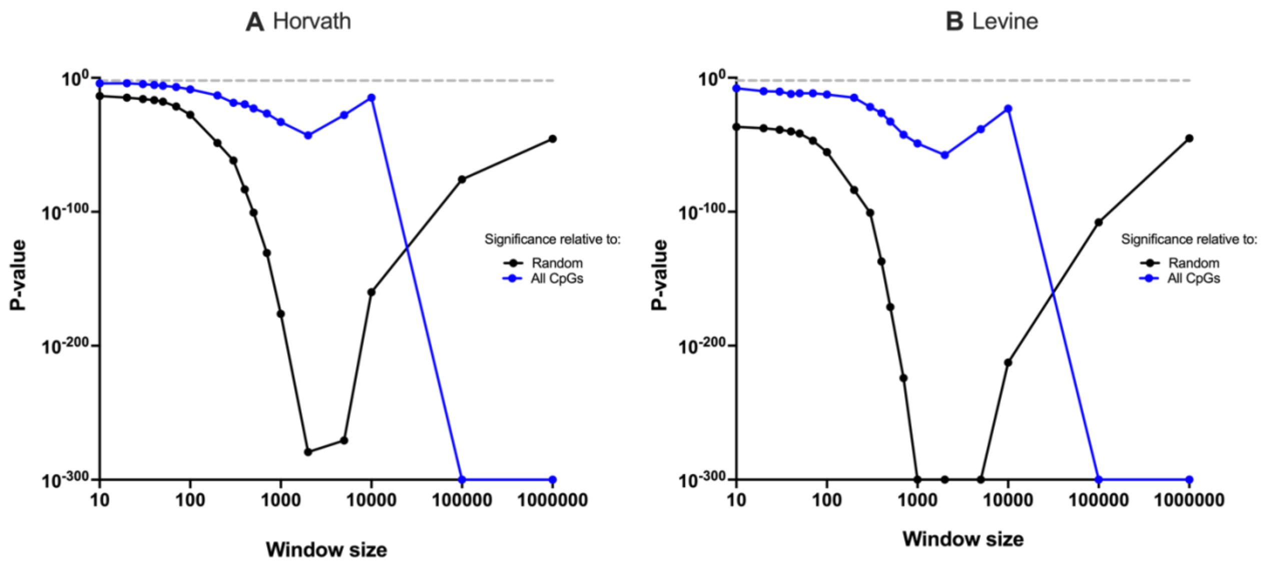

Figure S1. P-values for enrichment tests of G4s at A) Horvath and B) Levine human ageing clocks. Dotted line at p=0.01 for reference.

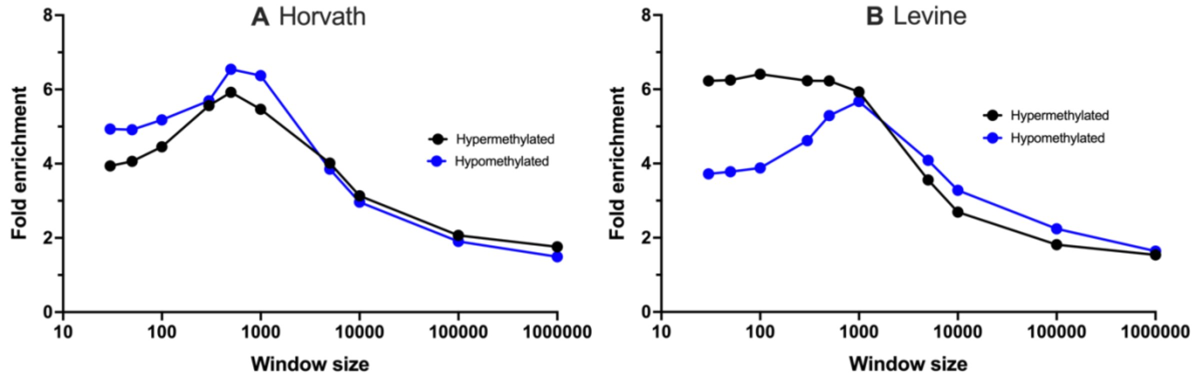

Figure S2. Enrichment of G4s at A) Horvath and B) Levine ageing clock CpGs that become hyper- or hypomethylated with age.

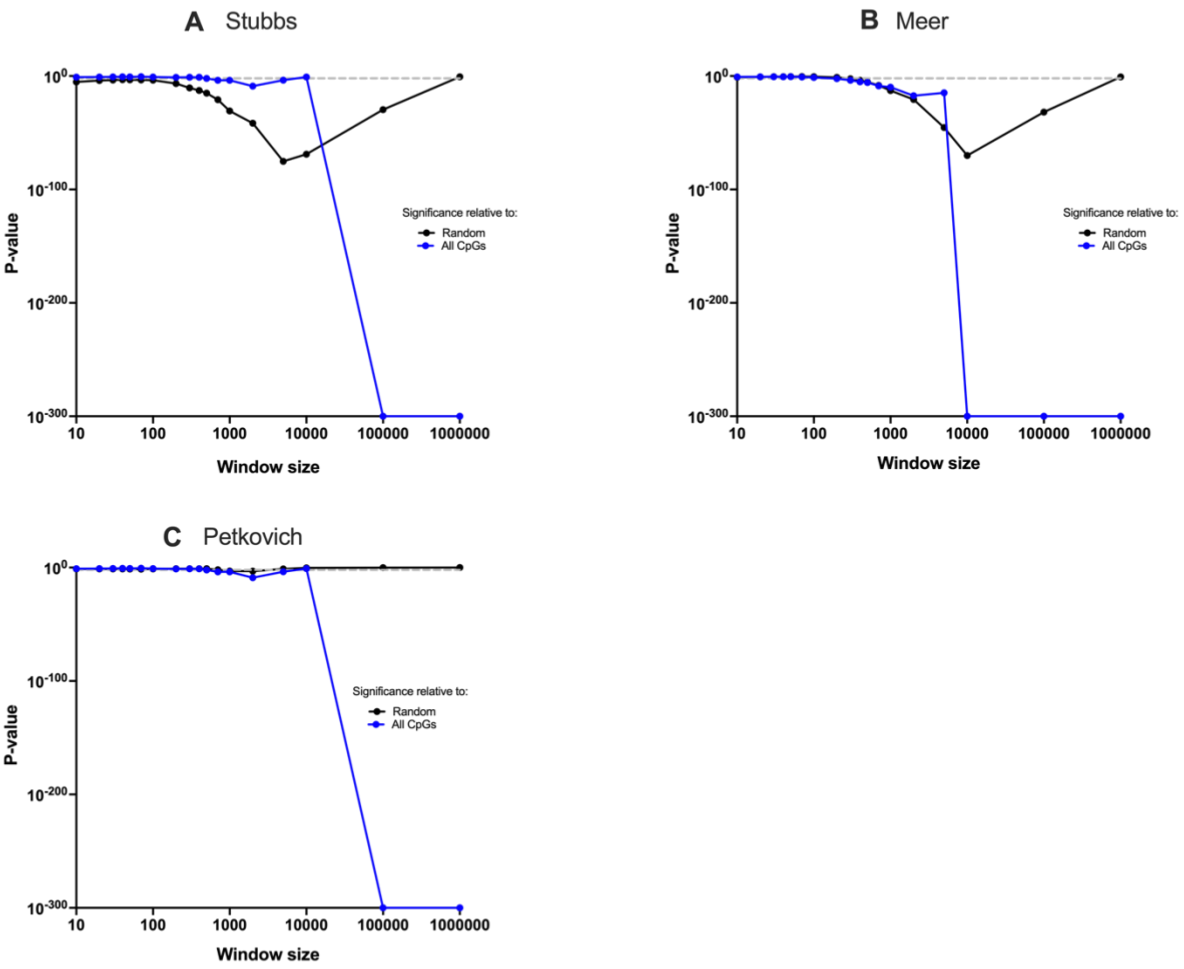

Figure S3. P-values for enrichment tests of G4s at A) Stubbs, B) Meer and C) Petkovich ageing clocks. Dotted line at p=0.01 for reference.

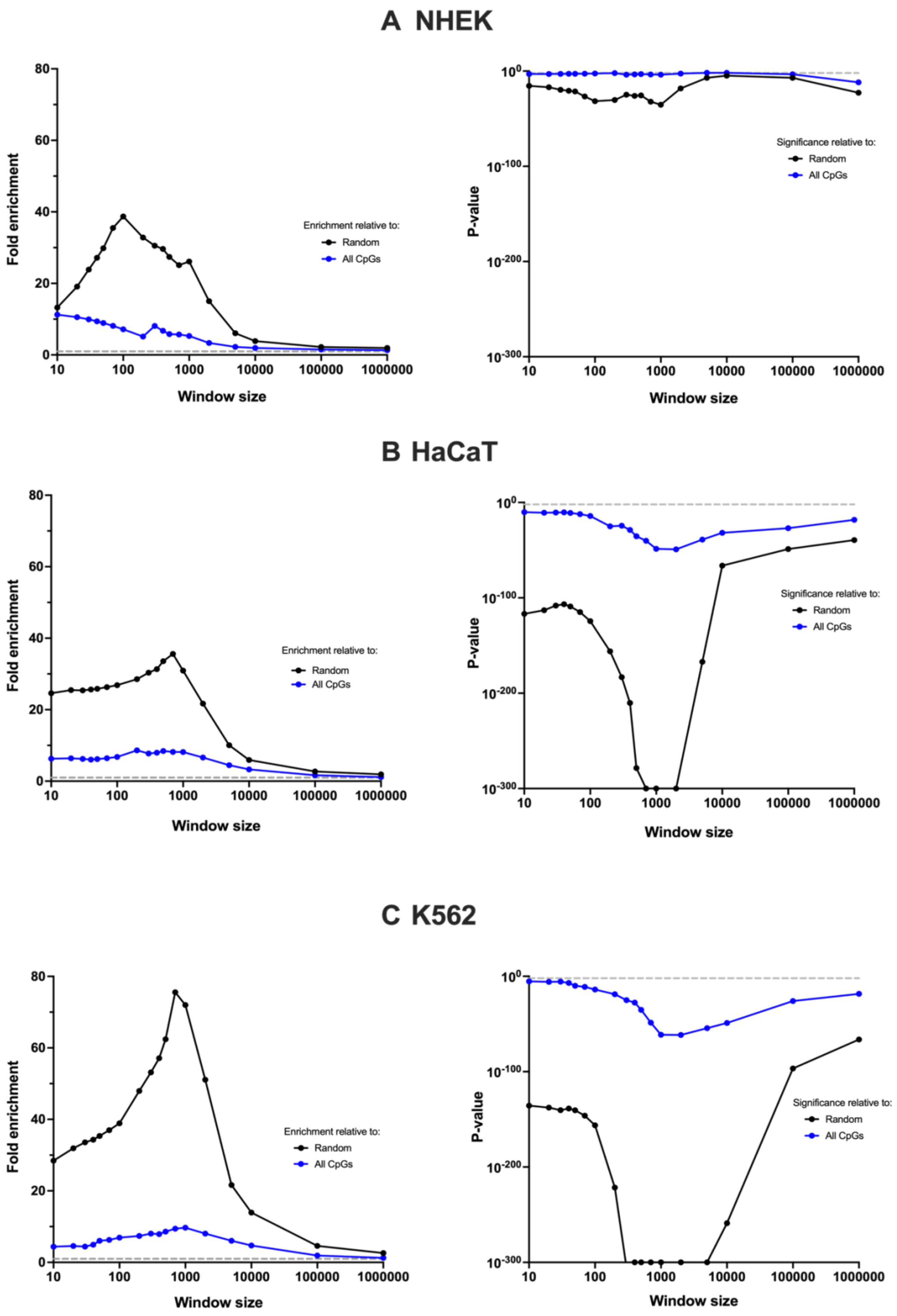

Figure S4. Fold enrichment and statistical significance of G4s around Horvath ageing clock CpGs. G4 coordinates were obtained from BG4-ChIP experiments in A) NHEK, B) HaCaT and C) K562 cells. Dotted line at foldenrichment =1 and p-value=0.01 for reference.

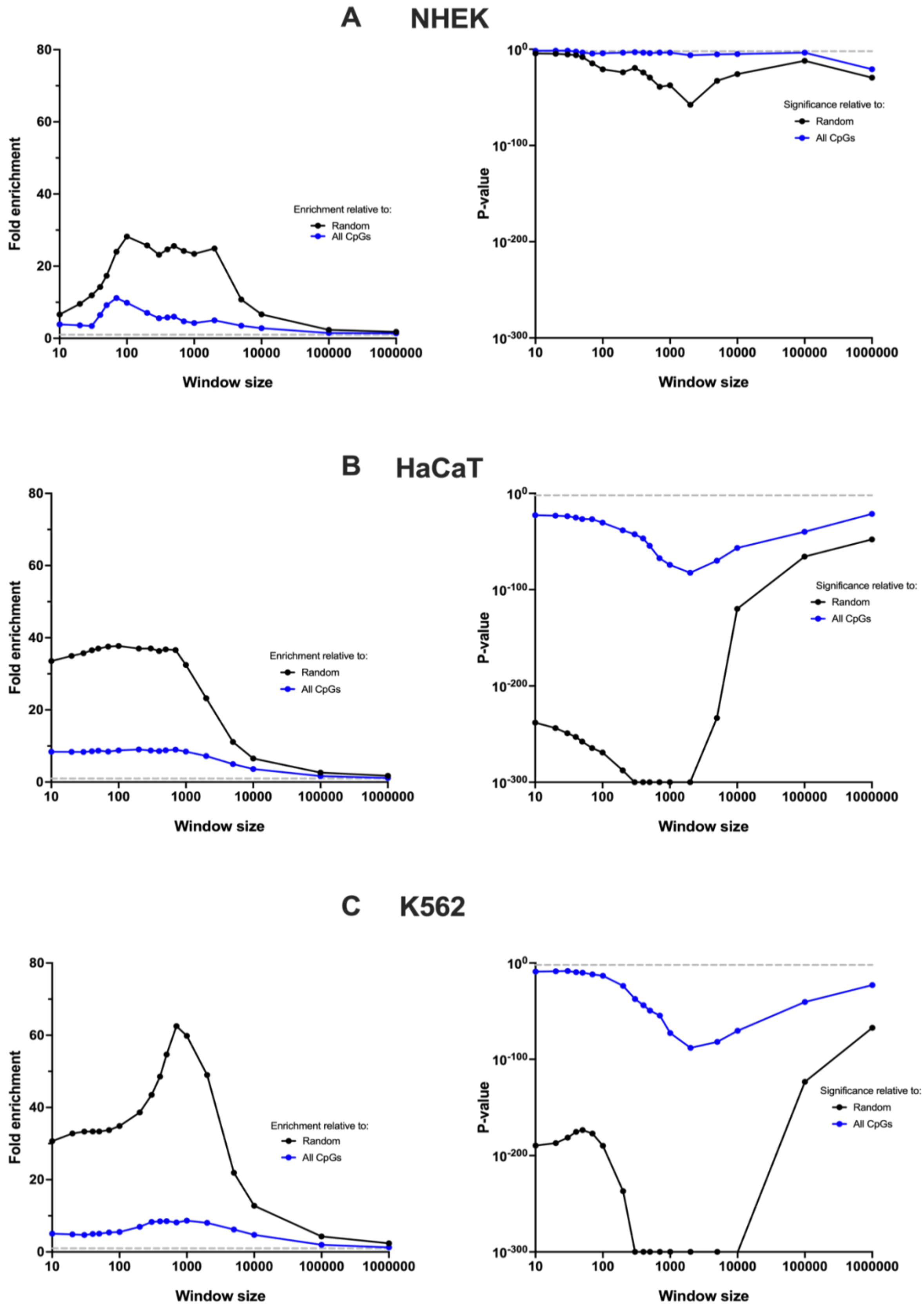

Figure S5. Fold enrichment and statistical significance of G4s around Levine ageing clock CpGs. G4 coordinates were obtained from BG4-ChIP experiments in A) NHEK, B) HaCaT and C) K562 cells. Dotted line at foldenrichment =1 and p-value=0.01 for reference.

Table S1 - Comparison of G4 enrichment at murine ageing clocks with and without random sampling

**Window size (bp)**	**Meer**	**Meer sampled**	**Stubbs**	**Stubbs sampled**	**Petkovich**	**Petkovich sampled**
10	1.266600595	1.395348837	2.137265136	2.595155709	1.68411	1.290323
50	1.071241488	1.411079588	1.808966636	2.065469293	1.702308	1.401869
100	1.116058508	1.559121032	1.805344535	1.983995168	1.679104	1.219512
500	1.649907712	1.784517172	2.268597901	2.357960925	1.34373	1.601423
1000	1.726744797	1.724427112	2.365559251	2.320901619	1.693543	2.1875
10000	1.555206906	1.568476302	1.739769379	1.750721348	0.988199	1.026616
1.00E+05	1.268608707	1.262819813	1.318043275	1.32873031	0.83681	1.015419
